# Development and Usability Assessment of a Health Education Conversational Agent for Patients With Gastric Cancer: Action Research Study

**DOI:** 10.2196/82746

**Published:** 2026-09-10

**Authors:** YiChen Kang, YaMin Yan, TianXiao Wang, ZhengHong Yu, Yan Hu, ZhiXun Wang, JiYang Zhang, Jos M Latour, YuXia Zhang

**Affiliations:** 1 Department of Nursing, Zhongshan Hospital, Fudan University, Fenglin Road 180, Shanghai, China, 86 64041990; 2 Planning and Management Center, Zhongshan Hospital, Fudan University, Shanghai, China; 3 Big Data and Artificial Intelligence Center, Zhongshan Hospital, Fudan University, Shanghai, China; 4 School of Nursing and Midwifery, University of Plymouth, Plymouth, United Kingdom

**Keywords:** health education, generative artificial intelligence, digital health, user-centered design, action research

## Abstract

**Background:**

To provide patients with gastric cancer with adequate health education information for effective overall management is crucial, while traditional manners exposed certain challenges. Conversational agents have increasingly been adopted for health care use to provide innovative solutions for patient education.

**Objective:**

This study aimed to develop a health education embodied conversational agent to focus on gastric cancer disease using an action research approach and test its accuracy, usability, and user experience among patients and other related stakeholders.

**Methods:**

The AI-guided conversational agent was developed based on the OpenMEDLab 2.0 foundation model and the Retrieval-Augmented Generation (RAG) architecture. A 4-phase action research approach was adopted to implement this system at a gastric cancer center in China. Diagnose and plan phase used participatory observation and in-depth interviews to explore current health education models and patients’ needs for health education. Act and implement phase was used to develop and deploy the conversational agent. Evaluate phase comprised 3 rounds of alpha testing to assess accuracy and RAG knowledge hit rate, and 1 round of beta testing to assess the usability and relevance. Reflect phase conducted in-depth interviews to gain insights into users' experiences. Data collection was conducted from September 2023 through April 2025. Participants include patients, clinical nurses, nursing managers, surgeons, clinical psychologists, and dietitians. Thematic analysis and multiple-group chi-square tests were performed, respectively, for qualitative and quantitative data. A 2-sided *P* value of <.05 was considered statistically significant.

**Results:**

A total of 44 patients, 13 nurses, 3 nursing managers, 2 surgeons, 1 clinical psychologist, and 1 dietitian were recruited during the study procedure. Favorable outcomes in terms of accuracy and usability were achieved. The accuracy of the agent in 3 rounds was 67% (37/55), 71% (44/62), and 82% (31/38), respectively; RAG knowledge hit rates reached 86% (47/55), 98% (61/62), and 100% (38/38). Significant differences (*P*<.01) in RAG knowledge hit rates were observed. The mean chatbot usability questionnaire score was 91.9 (SD 3.6), while the mean content relevance score was 3.75 (SD 0.9). Three themes of user experiences were identified: perceived usefulness, ease of use, and intention to use, revealing potential in reducing staff workload and reinforcing patient education.

**Conclusions:**

This study provided insights into how the action research approach can inform the development and usability assessment of a gastric cancer health education conversational agent, also illustrating the value of RAG technology. Additional assessments and improvements are warranted to confirm the effectiveness and safety.

## Introduction

### Background

Gastric cancer is the most common gastrointestinal tumor worldwide, ranking as the fifth most prevalent cancer and the fifth leading cause of cancer death globally [1]. Decreases in 1 or more domains (physical, role, social, etc) of functioning and an increase in negatively associated symptoms (dysphagia, reflux, etc) were found perioperatively among the patients with gastric cancer [2]. Furthermore, if patients are unfamiliar with managing these symptoms or lack adequate disease information, these problems may worsen. In addition to the impact of symptoms on the human body, related economic costs and psychological burdens also affect patients’ recovery [3]. Therefore, it is crucial to provide patients with gastric cancer with adequate health education information for effective overall management.

Patients and their families frequently encounter difficulties in accessing reliable and comprehensible disease-related information [4]. This gap is primarily due to the limitations of traditional health education methods, such as pamphlets, verbal instructions, and infrequent consultations, which often fail to meet the dynamic and personalized needs of patients with gastric cancer. Besides, these kinds of health education methods often include too much content with dense text, which may lead to information overload. Patients will feel confused when first facing a large amount of information and even more anxious and fearful with diagnosis and treatment. Patients with gastric cancer also face unique challenges due to the complexity of the surgery, subsequent symptoms, and perioperative care. Current health education resources and methods are insufficient to meet the demand for personalized, real-time responses, which proves to be a catalyst for innovative solutions.

With the development of technology, some patients seek online medical information when they feel uncertain or concerned. Nevertheless, such behavior sometimes may entail potential risks such as exacerbating anxiety and inappropriate self-management due to unverified information [5]. Digital health interventions, particularly conversational agents—such as chatbots or virtual assistants, offer promising solutions. Research has proved that conversational agents had the potential to significantly improve patient care by enhancing symptom management, supporting self-management, and providing patient support [6-8]. Gomaa et al [9] developed an SMS text messaging–integrated and chatbot-interfaced self-management program for patients with gastrointestinal cancer undergoing chemotherapy. The authors noted that the omission of natural language processing (NLP) capabilities restricted deeper interactive engagement, and future research should explore integrating NLP to enhance responsiveness to diverse patient expressions. Naseri et al [10] examined the effectiveness of 2 AI chatbots, Sider Fusion AI Bot and Perplexity AI, in improving patient outcomes, alleviating anxiety, and promoting informed decision-making, highlighting the importance of tailored communication styles to enhance patient engagement and outcomes. Thus, an AI-based conversational agent is suggested to use in our study.

However, generic and unverified AI-based conversational agents may pose potential health risks such as delivering inaccurate information that seems convincing [11]. Their integration into clinical practice is sometimes hindered by inherent limitations, and most refers to the phenomenon of “hallucinations” and unclear boundaries of responsibility and ethics [12]. A growing number of AI-driven platforms have been developed for high-prevalence malignancies, such as breast, prostate, and lung cancers, but rigorously designed conversational agents specifically tailored for gastric cancer remain scarce [13]. In the high-stakes context of gastric cancer care, where erroneous dietary or postoperative advice can lead to severe clinical complications, ensuring information fidelity is paramount. RAG has emerged as a robust architectural solution to mitigate these risks [14]. Zhou et al [15] developed a Chinese gastrointestinal disease chatbot and demonstrated the innovative potential of Retrieval-Augmented Generation (RAG) technology. The retrievable knowledge functions as a form of nonparametric memory that is easily updatable, capable of incorporating extensive long-tail knowledge, and suitable for encoding confidential information. Consequently, as the landscape of AI-based conversational agents continues to expand, the focus of development must transcend mere conversational fluency. Clinical safety must remain the cornerstone of AI deployment to ensure that these intelligent agents serve as a secure bridge between complex medical knowledge and the patient’s daily recovery.

Based on the clinical background and gap, 4 research questions are discussed in this study as follows: (1) How to explore patients’ needs and suggestions on the current gastric health education mode? (2) How to use large language models (LLMs) and RAG to develop a conversational agent for patients with gastric cancer? (3) How to evaluate the performance of the agent in terms of accuracy and usability? (4) How to reflect the user experience among patients and other related stakeholders regarding the conversational agent as a tool for patient education?

### Objectives

The aim of this study was to develop and implement an AI-guided conversational agent for patients with gastric cancer. We adopted an action research approach to gain insights from patients and stakeholders, develop the conversational agent, evaluate its usability and accuracy, and gather user feedback by using qualitative and quantitative methods.

## Methods

### Study Design

An action research design was adopted, which is a collaborative, democratic approach and process where research participants are collaborators rather than participants [16,17]. Unlike conventional approaches, action research emphasizes collaborative knowledge building and social change to develop contextually relevant interventions [18]. It has been applied by health care professionals to improve both patient experience and working conditions of those who deliver care [19,20]. The implementation of digital health interventions presents unique challenges in contrast to other studies [21]. To gain the most from digital health products such as conversational agents in our study, we should clearly know what is really needed in practice [20]. Co-design or collaboration with all stakeholders provides effective means for developing and implementing digital health interventions that suit the needs of the end users [22].

**Figure 1.**
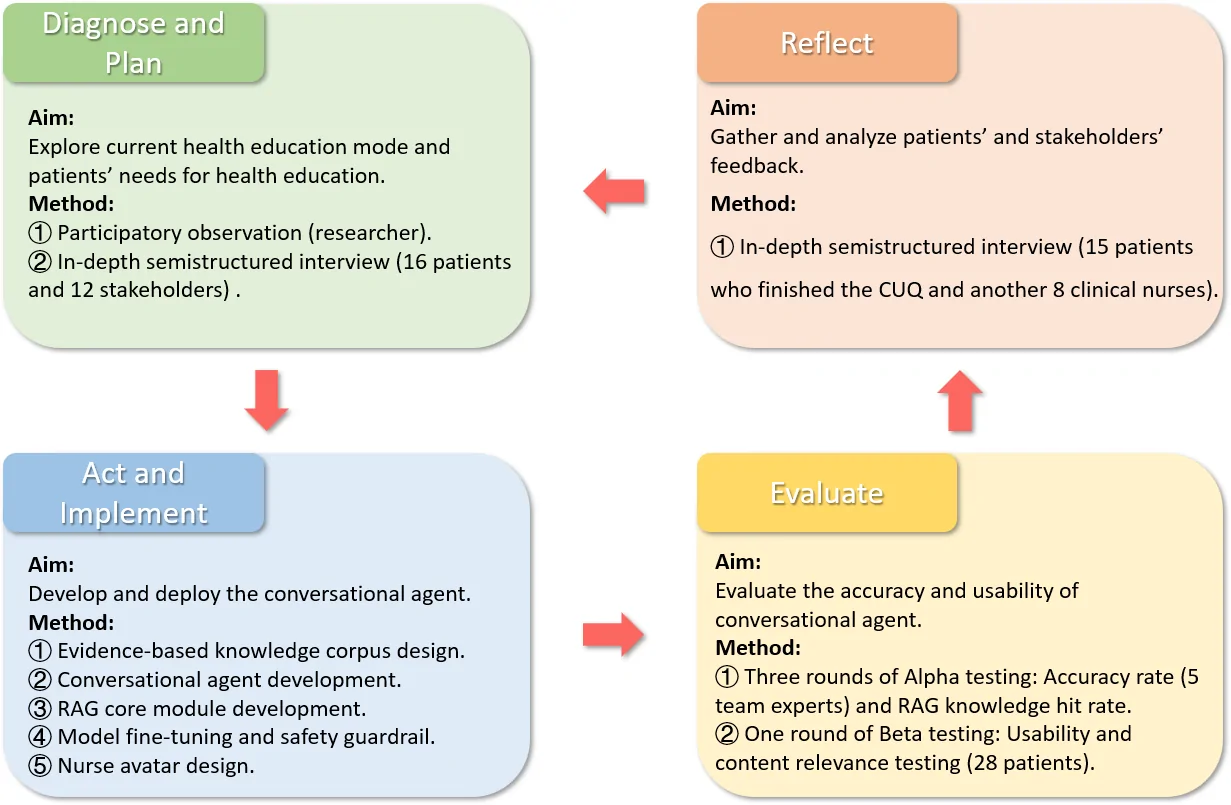
Action research cycle illustrating the iterative development of a health education conversational agent for patients with gastric cancer in this study. CUQ: chatbot usability questionnaire; RAG: Retrieval-Augmented Generation.

Action research emphasized that each study should adapt the action research framework according to its specific research aim and content. Our study followed an iterative process adapted from Lewin’s [23] 4-step cycle of action research (Figure 1). The reporting guideline and checklist (Checklist 1) have been used to report our study [24].

### Action Research Team

The multidisciplinary team was composed of 3 advanced nurse specialists, 2 surgeons, 2 researchers, and an information technology engineer. Each team member played a distinct and essential role in the development and implementation of the health education conversational agent. The advanced nurse specialists and surgeons who had 7-20 years of experience in the gastrointestinal field brought rich clinical experience in gastric perioperative care and patient education. They were responsible for identifying key health education weaknesses and constructing a knowledge base for the conversational agent. The researchers led the overall research design, data collection, and analysis process. The information technology engineer was responsible for conversational agent development, program coding, and other informatics work. The action team functioned as a partnership, characterized by shared decision-making and collaborative effort throughout all phases of the research.

### Setting and Participants

This study was conducted at the Gastric Cancer Center of Zhongshan Hospital in Shanghai, China, a facility known for its advanced medical care devices and patient-centered care delivery. Patients with gastric cancer and relevant stakeholders were involved in this action research study.

### Action Research Process

Four phases were designed to realize the triangulation of research by involving multiple participants and combining qualitative and quantitative approaches [25].

#### Phase 1: Diagnose and Plan

The first step of the action research is diagnosing and planning, which means researchers should thoroughly understand the needs of the target population and the problems for the current health education model. Therefore, participatory observation and in-depth interviews were used in the first stage of this action research.

By engaging in participatory observation, researchers were able to directly depict the structure, effectiveness, and limitations of clinical health education practices of participatory observation for patients with gastric cancer [26]. Within a week in September 2023, the researcher participated as a clinical nurse in the gastric cancer center, observing the daily workflow and content of the health education for patients. Data collection included multiple methods: direct observation, audio recordings, and document analysis. The key aspects of the observation included (1) timing and triggers of health education delivery, (2) content and themes of health education, (3) delivery and interaction methods of health education, and (4) patients’ observable cognitive, emotional, and behavioral responses to health education. Face-to-face semistructured interviews with 16 patients and 12 stakeholders were conducted after the participatory observation. These interviews aimed to explore the existing problems in gastric cancer health education and to identify stakeholders’ needs and expectations for the development of a health education conversational agent. The in-depth interview outline is tailored for each participant (Table S1 in Multimedia Appendix 1).

#### Phase 2: Act and Implement

Action research team collaboratively developed the conversational agent based on results from both participatory observation and in-depth interviews. An evidence-based knowledge corpus was constructed based on multiple sources of professional evidence. The AI-guided conversational agent was developed based on the OpenMEDLab2.0 foundation model and the RAG architecture. To enhance user engagement and provide a more human-like interaction experience, a nurse avatar was integrated into the chatbot interface. Electronic materials are presented in Multimedia Appendix 2.

#### Phase 3: Evaluate

Three rounds of alpha testing and 1 round of beta testing were conducted to evaluate the accuracy and usability of the conversational agent. Accuracy rate and RAG knowledge hit rate were measured in the alpha testing. In total, 55, 62, and 38 questions from the knowledge corpus were randomly selected for team experts to assess the accuracy of the answers in 3 rounds. Sample question used for expert evaluation is shown in Table S2 in Multimedia Appendix 1. A total of 28 patients were recruited to finish the chatbot usability questionnaire (CUQ) to assess the chatbot usability. They also finished a postconversation evaluation of content relevance, rating how well the chatbot’s responses matched the questions they asked.

#### Phase 4: Reflect

A total of 15 patients in the usability testing participated in the in-depth interview to deeply explore real experience and suggestions for the conversational agent. Besides, another 8 nurses who worked in the gastric cancer center were also invited to give their views on the new health education platform. The interview outline is shown in Table S3 in Multimedia Appendix 1. The results of qualitative study were used to guide the next cycle of action research.

### Participant Recruitment and Data Collection

Data collection was conducted from September 2023 through April 2025 across 4 sequential phases. Participants were recruited using convenience sampling from the gastric cancer center of Zhongshan Hospital. Eligible participants were identified by action research team members including patients, clinical nurses, nursing managers, surgeons, clinical psychologists, and dietitians (recruitment criteria in each phase are shown in Table S4 in Multimedia Appendix 1).

Qualitative interviews were recorded using a digital voice recorder with participants’ permission. Audio recordings were transcribed verbatim into text immediately after each interview. Data collection ceased until no new themes or meaningful information emerged from subsequent interviews. Inpatients who used the conversational agent were invited to finish the validated CUQ on discharge day. CUQ is a chatbot-specific usability questionnaire that is comparable with the Systems Usability Scale [27]. It consists of 16 balanced questions related to different aspects of chatbot usability. Odd-numbered questions relate to positive aspects of the chatbot, and even-numbered questions relate to negative aspects. All 16 questions are scored using a 5-point Likert-type scale. Each item was scored on a 5-point Likert scale, ranging from 1 (strongly disagree) to 5 (strongly agree). Scores are calculated out of 100. Besides the CUQ, the conversational agent was also tested by 1 more question about the content relevance. The additional question “Do you think the responses provided by the conversational agent appropriately match your question?” was also rated on a 5-point Likert scale as CUQ, and this score was analyzed separately. Face validity was assessed among 2 patients and 1 nurse.

The accuracy and RAG knowledge hit rate are 2 critical metrics for analyzing the performance of question-answering systems [28]. The accuracy rate was evaluated by 5 team experts (3 advanced nurse specialists and 2 surgeons) independently; each response was rated as correct or incorrect, and it was considered accurate if at least 4 of the 5 experts judged it to be correct. Overall interrater agreement across all 3 rounds of evaluation was calculated to verify the consistency of the expert assessments. The RAG knowledge hit rate determines whether the system can accurately retrieve relevant medical information from the knowledge base, preventing answer errors caused by missing knowledge or retrieval failures while mitigating the risk of large-model “hallucinations.” It was calculated directly by the system. We combined both automated retrieval metrics and manual assessment to offer practical and adequate evaluation results.

### Statistical Analysis

#### Qualitative Data Analysis

NVivo (version 20; QSR International) and content analysis approach [29] were used to identify themes and subthemes from the participatory observation and interview data. Two independent reviewers (YCK and YMY) launched the initial coding by immersing themselves in the data and reading the interview transcripts word by word. A tree diagram was used to organize the subthemes into themes. A discussion was held after the initial coding to resolve any discrepancies until a consensus was reached to ensure the consistency and completeness in the final results.

#### Quantitative Statistical Analysis

EXCEL and SPSS software (version 26.0; IBM Corp) were used to complete the quantitative data analysis. A CUQ calculation tool, a Microsoft Excel spreadsheet, which was developed by the researchers from Ulster University, is available for the easy calculation of the CUQ scores [30]. Outcome measures, including accuracy and usability, were evaluated through 3 calculation formulas: (1) Accuracy = (Number of correctly answered questions evaluated by health care experts/Total number of questions) × 100%. (2) RAG Knowledge Hit Rate = (Number of questions successfully matched with answers from the knowledge base/Total number of questions) × 100%. (3) $CUQ=((\Sigma_{n=1}^{m}2n-1)-5)+(25-(\Sigma_{n=1}^{m}2n))\times 1.6$

Descriptive statistics are presented with mean (SD), median (IQR), or frequencies (percentages), as appropriate. Comparisons among 3 rounds of accuracy rate and RAG knowledge hit rate were conducted using multiple-group chi-square tests. The interrater agreement was evaluated using the Fleiss κ. A 2-sided *P* value of <.05 was considered statistically significant.

### Ethical Considerations

The use of AI-based conversational agents in health care raises important ethical issues, particularly regarding data privacy, transparency, and patient autonomy. The conversational agent was designed to support, rather than replace, professional medical advice, in order to minimize the risk of overreliance on AI-generated information. The system repeatedly emphasizes that qualified health care professionals’ advice should be regarded as the final authority. Absolute or definitive language is deliberately avoided in responses, especially in certain sensitive topics such as diagnosis and treatment. Besides, conversations were anonymized and stored in encrypted files accessible only to the research team.

This study was approved by the Medical Ethical Review Board of Zhongshan Hospital Fudan University (B2022-613R). Individual informed consent was obtained from all participants, whether in the qualitative interviews or the quantitative researches. Participants were also informed that their personal identifiers were removed from the research database and they were voluntary to withdraw from the project at any time without giving a reason. This research complies with the Declaration of Helsinki and conforms to the data protection guidelines outlined by the local governing bodies.

## Results

### Phase 1: Diagnose and Plan

#### Participatory Observation Findings

The observational data revealed the traditional health education workflow, including the following: (1) patients typically receive health education at 4 key time points: upon admission, before surgery, after surgery, and at discharge, and if they have problems, they would ask health care staff anytime; (2) health education content covered topics such as preoperative preparation, postoperative recovery, dietary management, medication guidance, follow-up arrangements, complication prevention, and psychological support; (3) delivery methods primarily rely on oral instructions, printed materials, and video-based content, and educational videos were played on a continuous loop in public areas of the ward for inpatients, and some nurses use special ways such as teach-back methods to assess the understanding of patients; and (4) patients usually face information overload.

#### In-Depth Interview Findings

Sixteen patients with gastric cancer and 12 stakeholders were recruited in semistructured interviews. Detailed information is shown in Tables 1 and 2.

**Table 1.** Characteristics of patients with gastric cancer who were recruited to explore the existing problems in gastric cancer health education and needs for the development of a health education conversational agent (N=16).

Patients, sex	Age, years	Education level	Marital status	Career status
P1: female	61	Senior high school	Married	Retired
P2: female	77	Senior high school	Married	Retired
P3: female	44	Bachelor’s degree	Married	Worker
P4: male	56	Master’s degree	Married	Teacher
P5: female	77	Junior high school	Married	Retired
P6: male	51	Senior high school	Married	Farmer
P7: female	41	Bachelor’s degree	Married	Worker
P8: male	77	Senior high school	Married	Retired
P9: female	81	Senior high school	Married	Retired
P10: female	47	Bachelor’s degree	Married	Worker
P11: male	45	Bachelor’s degree	Married	Worker
P12: male	78	Senior high school	Married	Retired
P13: male	59	Senior high school	Married	Retired
P14: female	38	Bachelor’s degree	Married	Worker
P15: male	25	Bachelor’s degree	Single	Worker
P16: male	75	Senior high school	Married	Retired

**Table 2.** Characteristics of stakeholders who were recruited to explore the existing problems in gastric cancer health education and needs for the development of a health education conversational agent (N=12).

Stakeholders, sex	Age, years	Education level	Role
S1: female	29	Bachelor’s degree	Nurse
S2: female	34	Bachelor’s degree	Nurse
S3: female	31	Bachelor’s degree	Nurse
S4: male	28	Master’s degree	Nurse
S5: female	35	Bachelor’s degree	Nurse
S6: male	45	Master’s degree	Nursing manager
S7: female	52	Master’s degree	Nursing manager
S8: male	39	Master’s degree	Nursing manager
S9: female	47	Doctorate	Surgeon
S10: female	50	Doctorate	Surgeon
S11: male	42	Doctorate	Clinical psychologist
S12: male	40	Doctorate	Dietitian

Four key findings emerged from the interviews, including fragmented and inconsistent health education content, limited accessibility and personalization of education delivery, unmet needs for timely and interactive support, and expectations and concerns for new, technology-supported education manners (Table 3). Detailed analysis of each quote is provided in Text 1 in Multimedia Appendix 1.

**Table 3.** Themes identified from the in-depth interview to explore the disadvantages of current health education mode and expectations for new education manners.

Themes	Sample quotes from participants
Fragmented and inconsistent health education content	“I listened very carefully when the nurse was giving the health education, but afterward, I could only rely on the printed leaflet they gave me to recall the information...I feel like I didn’t remember a lot of it.” [P1]
Limited accessibility and personalization of education delivery	“The booklet they gave me was the same for everyone. Some parts didn’t apply to me at all, while the things I really cared about were not explained in detail.” [P4]
Unmet needs for timely and interactive support	“I was very anxious before the surgery...Although the preoperative education helped alleviate some of my anxiety and answered some of my questions, I felt it wasn’t enough. However, I was also worried about bothering the doctors and nurses too much.” [P3]
Expectations and concerns for new, technology-supported education manners	“We’re living in the information age now, and I hope there can be more intelligent methods and approaches for delivering health education to me.” [P6] “AI has indeed developed rapidly and is widely applied now, but we must also use it with caution. It should only be introduced to patients after confirming that it is reliable, practical, and provides accurate responses.” [S8]

### Phase 2: Act and Implement

#### Evidence-Based Knowledge Corpus Development

As a prerequisite for developing the conversational agent, a corpus was established to provide a reliable and clinically validated knowledge foundation for response generation. Based on the results of phase 1, the action research team first identified 9 core knowledge domains for gastric cancer health education: disease knowledge, diagnosis, prevention, treatment, nutrition, nursing care, recovery, follow-up, and hospitalization procedures. Guided by these domains, 2 researchers (YCK and YMY) searched data from structured and unstructured resources, including clinical practice guidelines, scientific literature, hospital patient education materials, clinician-compiled frequently asked questions, and other authoritative resources. Detailed search strategies and data sources are provided in Table S5 in Multimedia Appendix 1. After removing duplicates, advanced nurse specialists and surgeons of our gastric cancer center screened eligible evidence and transformed the curated knowledge into structured question and answer (Q&A) pairs. The Q&A knowledge corpus would be integrated into the conversational agent as its knowledge base to support retrieval-augmented response generation. This corpus will be continuously maintained by chief physicians and nurses from the gastric cancer center, with per-week updates to incorporate the latest clinical evidence and practice guidelines.

#### Conversational Agent Development and Functions

**Figure 2.**
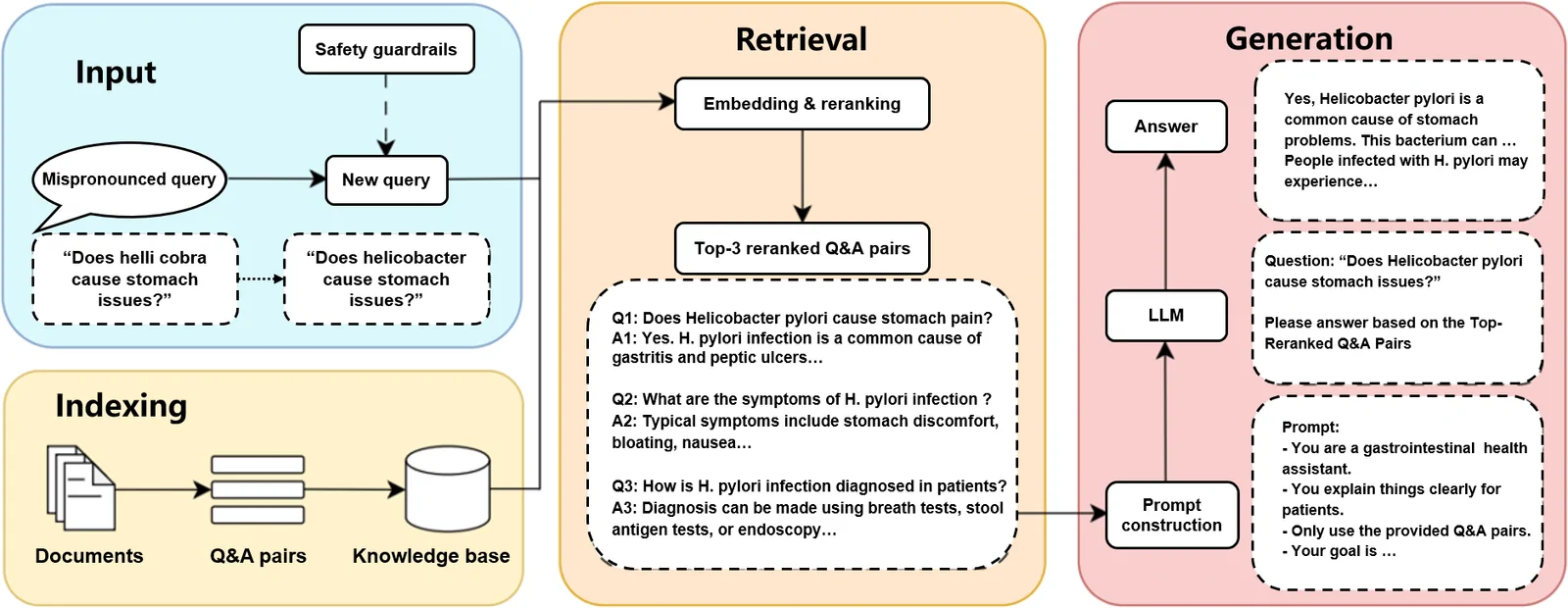
A detailed operation illustration example of the conversational agent based on the OpenMEDLab2.0 foundation model and the Retrieval-Augmented Generation architecture. LLM: large language model; Q&A: question and answer.

This conversational agent constructed a question-answering service system based on the OpenMEDLab2.0 foundation model and the RAG architecture (Figure 2). As a domain-specific LLM, the OpenMEDLab2.0 foundation model was developed based on InternLM and achieved professional capability through a 3-stage progressive medical domain–training pipeline: (1) continual pretraining on medical literature, clinical guidelines, and diagnostic cases processed by third-generation data-cleaning technology, using masked language modeling and sentence order prediction tasks with BF16 mixed-precision training; (2) supervised fine-tuning on over 300,000 medical instruction datasets via low-rank adaptation for parameter-efficient training; and (3) 3 rounds of online reinforcement learning from human feedback (RLHF) to optimize response safety and clinical relevance. This training process enables the model to better capture medical semantic relationships and represent domain-specific knowledge, thereby improving its ability to interpret complex medical queries and generate structured responses [31].

For this study, the RAG architecture was specifically tailored to integrate with the OpenMEDLab2.0 foundation model. The RAG knowledge base was constructed by the evidence-based knowledge corpus developed in the last procedure. The retrieved information was injected into the input layer of the OpenMEDLab2.0 model as structured context. Specifically, the retrieved medical knowledge was first converted into vector data and then subjected to relevance matching via a rerank fine-ranking model to obtain the top 3 most relevant items. The most relevant knowledge was input into the OpenMEDLab2.0 model as a prompt to generate the final output. This method helps reduce the “hallucination” issues caused by knowledge limitations in pretrained large models and significantly improves the timeliness and accuracy of the model’s responses, as the knowledge base mounted by RAG is synchronously updated with the latest clinical guidelines and expert consensus.

The workflow of the conversational agent begins with a patient’s inquiry and consists of four steps:

1. Step 1: When a patient submits a query, the system first corrects any errors in speech recognition through hotword revision to convert misrecognized parts into accurate content. Subsequently, the user query is processed and analyzed by the safety guardrails module, which is specifically designed for sensitive content filtering. This module detects inappropriate information, including pornography, gambling, drugs, violence, and other high-risk content in patients’ questions. If any prohibited sensitive terms are identified, the system will directly refuse to generate a response. In addition, the same module will also conduct a safety review on the output answers generated by the large model; any response containing sensitive or risky content will be blocked accordingly. Only queries and corresponding outputs that fully pass the dual safety checks are allowed to enter the subsequent processing steps. If the patient’s question-answer interaction does not comply with safety standards, the system will refuse to respond; queries that pass the safety check will proceed to the next step.
2. Step 2: The retrieval module converts the identified core needs into vector representations using an embedding model. The embedding model maps textual information into a low-dimensional vector space, where semantically similar texts are positioned close to each other. The vector database contains medical knowledge curated by professional health care staff and precisely broken down into rich question-answer pairs, each of which is converted into a vector and stored in the database. First, all types of medical knowledge resources, including structured medical data, unstructured clinical documents, and medical education videos, were systematically organized and converted into a standardized Q&A pair format. For nontextual resources such as medical videos, key medical information was first extracted through manual annotation by clinical professionals and intelligent content recognition technology and then transformed into Q&A pairs that conform to clinical consultation scenarios. Subsequently, question augmentation was performed on the constructed Q&A pairs to improve the generalization ability of knowledge retrieval; specifically, techniques such as synonym replacement, sentence structure transformation, and clinical scenario extension were adopted to generate multiple extended question versions for each original question, ensuring that the knowledge base can effectively match various expression styles of patients’ queries. Finally, all the standardized Q&A pairs (including original and extended ones) were uniformly subjected to vectorization processing using an encoder. During the vectorization process, the medical semantic features of the Q&A pairs were fully extracted and converted into dense vectors, which were then stored in the vector database to support efficient dynamic retrieval of the RAG architecture. During retrieval, the system calculates the similarity between the patient’s query vector and the vectors of the question-answer pairs in the database to efficiently identify relevant candidates.
3. Step 3: The candidate question-answer pairs then enter the reranking module. This module evaluates and ranks them based on their relevance to the patient’s query using machine learning algorithms. The top 3 most relevant question-answer pairs are selected as input for the LLM.
4. Step 4: After retrieving the relevant information from the knowledge base, the LLM generates the final response. The reranked question-answer pairs are fed into the model according to a preconfigured prompt structure. This structured prompting, along with the top 3 matched answers, guides the model to generate responses that are accurate, professional, and aligned with ethical standards, which are then delivered to the patient.

The conversational agent was deployed on the intranet of Zhongshan Hospital with a Client/Server (C/S) architecture to ensure medical data security. The service is provided by a dedicated system: the client terminal includes a transparent LCD screen (for interaction) and a computer with an NVIDIA GeForce RTX 3090 graphics card (exclusively for digital human rendering), while the real-time inference service of the RAG-based large model is provided by a virtual machine on a computing power server equipped with Huawei Ascend 910B chips. Currently, it is available only in the hospital’s gastric cancer center and not deployed online or on mobile devices. Functions are mainly divided into 2 modes: frequently asked questions and free questioning. Patients can click on frequently asked questions to view preset questions and answers, or choose the free question mode to ask questions. When the virtual person responds, its voice and gestures will mimic those of a real person as much as possible with automatic speech recognition, text-to-speech, and lip-sync technology, thereby providing patients with a more realistic Q&A experience. Patients can adjust the volume by clicking the button on the right side of the screen.

#### RAG Core Module Development

The RAG system in our study was developed around a vectorized knowledge base and 2 core components, namely, the retriever and the generator [14]. The construction of a vector knowledge base involves transforming preprocessed text fragments and question-answer pairs into high-dimensional vectors, selecting a vector database that supports efficient semantic retrieval, importing vector data, establishing mapping relationships between vectors and question-answer pairs, configuring indexes to optimize retrieval speed, and adapting to real-time consulting needs.

The retriever module was developed using a retrieval strategy that combines sparse retrieval with a reranking model, with a primary focus on delivering the top-3 most accurate results. First, sparse retrieval based on keyword matching was used to locate core document segments and quickly generate a candidate set of 50‐100 items. Next, a reranking model adapted to the medical context was developed to perform deep ranking of the candidate results based on dimensions such as medical semantic relevance and clinical priority, precisely selecting the top-3 most relevant knowledge segments or question-answer pairs to ensure accurate alignment with core clinical knowledge. Finally, the top-3 results underwent a final validation step to filter duplicates and content exceeding specialty-specific safety boundaries, ensuring the uniqueness and safety of the output.

In the generator module, the retrieved question-answer pairs were used as contextual input to the OpenMEDLab2.0 foundation model, with explicit enforcement of medical ethics and specialty-specific safety boundaries. For complex queries, multiple knowledge segments were integrated to generate coherent responses, ensuring that the language remained both accessible and professionally accurate. The system also supported multiturn conversational memory, allowing historical consultation content to be referenced in order to optimize subsequent responses.

#### Model Fine-Tuning and Safety Guardrail

To ensure the safety of LLMs in our conversational agent, the system uses RLHF for model fine-tuning. RLHF enables the model’s behavior and outputs to better align with ethical norms and social values. Furthermore, through safety guardrail, incorporating interception, rewriting, and proxy-answering functionalities, the system identifies and blocks potential malicious prompts or erroneous outputs. This ensures that the system can respond reasonably when encountering latent ethical risks.

#### Nurses as the avatar of the conversational agent

**Figure 3.**
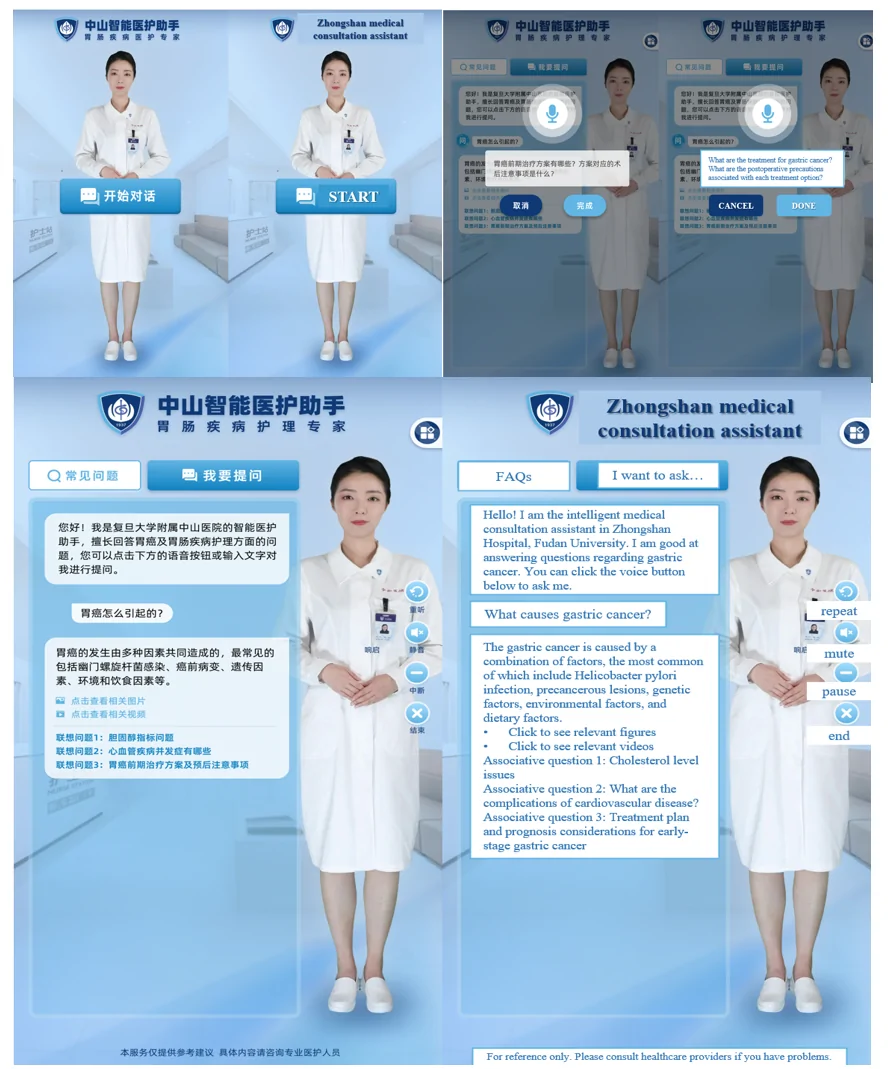
Interface of the health education conversational agent including the home page and chatting screen.

The chatbot featured a vividly embodied image—an approachable and empathetic digital avatar designed to resemble a compassionate nurse (Figure 3). This design choice is grounded in both emotional appeal and clinical relevance [32]. By adopting the image of a nurse, the chatbot conveys a sense of warmth, safety, and reliability that patients can easily relate to. Higher acceptance and increased adherence to treatment regimens were also found in some studies [33,34].

Both verbal and nonverbal behaviors were designed for the conversational agent. Verbal behaviors mainly include some specific language constructs such as politeness strategies, inclusive pronouns, and greeting and farewell rituals. Nonverbal behaviors mainly include pleasant facial expressions, lip movements, head nods, and hand gestures synchronized with speech. Such visual elements were proved to be beneficial for patients in establishing rapport and engagement with the chatbot [33].

### Phase 3: Evaluate

#### Alpha Testing

The accuracy of the agent in 3 rounds was 67% (37/55), 71% (44/62), and 82% (31/38), respectively; RAG knowledge hit rate reached 86% (47/55), 98% (61/62), and 100% (38/38). Significant differences (*P*<.01) in RAG knowledge hit rate were observed across 3 rounds of testing, while no significant differences (*P*=.30) were found in accuracy. Overall interrater agreement using the Fleiss κ (0.85; *P*<.01) was revealed. RAG knowledge hit rate was automatically evaluated by the system, and interrate reliability was not applicable.

#### Beta Testing (Usability Testing)

Twenty-eight participants were included in the chatbot usability testing. Characteristics of 28 patients are shown in Table 4.

**Table 4.** Characteristics of patients who used the conversational agent and finished the chatbot usability questionnaire (N=28).

Characteristics	Patients
Sex, n (%)	
Male	12 (43)
Female	16 (57)
Age (years), n (%)	
<40	2 (7）
40‐55	5 (18）
55‐70	8 (29）
>70	13 (46）
Hospitalization, days, n (%)	
<3	3 (11）
3‐10	20 (71）
>10	5 (18）
Marital status, n (%)	
Married	22 (79）
Single	2 (7）
Widowed	0 (0）
Divorced	0 (0）
Others	4 (14）
Medical insurance, n (%)	
Yes	26 (93）
No	2 (7）
Cancer disease, n (%)	
Yes	25 (89)
No	3 (11)

**Figure 4.**
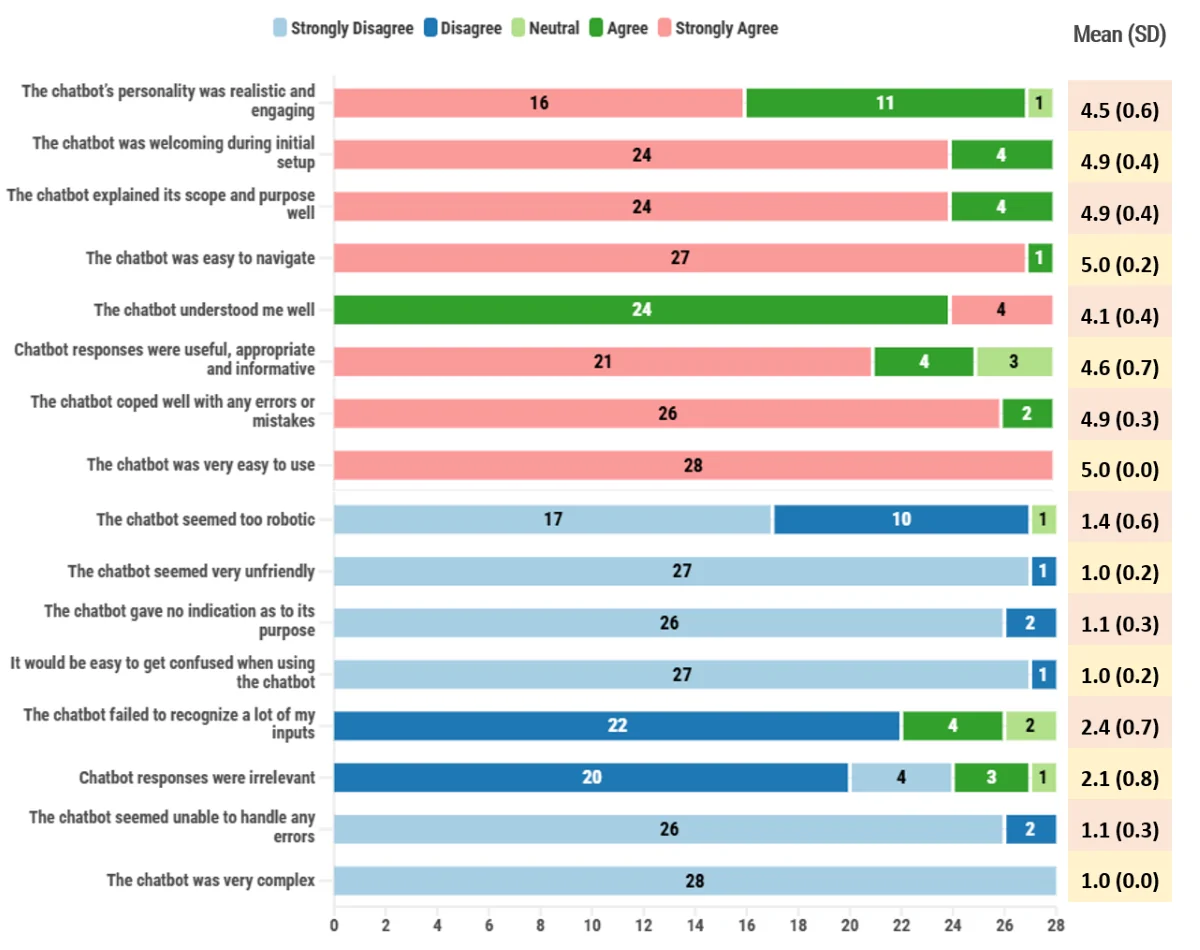
Patients’ choices and mean scores on each item of the chatbot usability questionnaire.

The mean CUQ score was 91.9 (SD 3.6), with a median score of 92.2 (range 79.7‐95.3) (Figure 4). Among the odd questions, statements “The chatbot was easy to navigate” and “The chatbot was very easy to use” received the highest mean scores which proved that this conversational agent was easy to use. The statement “The chatbot understood me well” got the lowest mean scores and showed that some understanding problems still existed in this system. Among the even questions, the statements “The chatbot seemed very unfriendly,” “It would be easy to get confused when using the chatbot,” and “The chatbot was very complex” got the lowest mean scores, while “The chatbot failed to recognize a lot of my inputs” got the highest mean scores. These indicated that input and output of this conversational agent still had some problems that may confuse participants. Each score of CUQ is detailed in Table S6 in Multimedia Appendix 1. Besides, the mean content relevance score was 3.75 (SD 0.9), with a median score of 4 (range 3‐4).

### Phase 4: Reflect

We recruited 15 patients (T1-T15) in the last phase who finished the CUQ and 8 nurses to do the in-depth interview to deeply explore the factors facilitating and hindering the conversational agent. Detailed characteristics of 8 clinical nurses are shown in Table 5.

Emerging themes from the qualitative study results were mapped on the 3 domains of the technology acceptance model (Table 6). Detailed information about the qualitative analysis is shown in Text 2 in Multimedia Appendix 1.

**Table 5.** Characteristics of clinical nurses who worked in the gastrointestinal center and participated in daily health education activity (N=8).

Clinical nurses, sex	Age, years	Career years	Education level
N1: female	27	5	Bachelor’s degree
N2: female	30	8	Bachelor’s degree
N3: male	34	11	Master’s degree
N4: female	26	4	Bachelor’s degree
N5: male	32	10	Bachelor’s degree
N6: female	29	7	Bachelor’s degree
N7: female	35	13	Bachelor’s degree
N8: male	31	9	Bachelor’s degree

**Table 6.** Themes identified to explore the factors facilitating and hindering the use of conversational agent.

Theme and subthemes	Representative quotes
Perceived Usefulness	“It was like having a knowledgeable companion by my side who could answer my questions at any time.” [T3] “Sometimes I asked a very simple question, but the answer is too long and the language is quite academic, which in turn made me feel burdened.” [T7] “We may not always be able to provide patients with comprehensive educational content, but the conversational agent helps us to supplement this and make up for any omissions in our work.” [N2]
Perceived Ease of Use	“I thought it would be difficult to use, but after the nurse showed me how, I learned it immediately. It wasn’t difficult at all.” [T10] “Do I have an accent? So when I use it, it always misunderstood what I’m saying, which seems to affect its usability a little.” [T8] “This conversational agent is indeed very convenient to use, and we don’t really need to teach patients how to use it.” [N5]
Intention to Use	“It feels like I’m really talking to a nurse, not just a machine...She looks kind and makes me feel more at ease when asking questions.” [T9] “I feel that this machine is really easy to use. It would be great if you could develop a mobile app so that I can ask questions when I get home if I don’t understand something.” [T6] “This conversational agent is very useful, but we still need to improve it to make it more mature and reliable.” [N1]

## Discussion

### Principal Findings

This study demonstrates how the action research framework can be a practical methodology to develop a health education conversational agent, comprising the diagnose and plan, act and implement, evaluate, and reflect phases. These 4 phases formed a continuous and iterative process in which the findings from each phase informed the subsequent stage. This user-centered approach enabled the systematic integration of patient needs, clinical expertise, technical implementation, and iterative evaluation, and offered an approach for developing digital health interventions that are both clinically relevant and acceptable to end users. By placing the diagnose and plan phase at the beginning of the process, researchers were able to identify real clinical requirements rather than technological assumptions and avoid certain barriers to adoption at the earliest stage. The act and implement phase realized outcomes that the conversational agent not only incorporated advanced technologies but also remained clinically practical and aligned with routine health care delivery by involving multidisciplinary stakeholders. The successful implementation of technologies such as RAG also relies on close collaboration between medical and informatics specialists. The evaluate and reflect phases constituted essential components of the iterative optimization process. Integration of quantitative and qualitative findings enabled developers to identify specific directions for system refinement and provided a foundation for subsequent iterative development. Overall, the action research–based development pathway in our study may offer opportunities for improving the sustained adoption of digital health interventions in real-world clinical settings through iterative, user-centered, multidisciplinary, and mixed methods principles.

### Clinical Implications

Health education is a cornerstone of therapy and recovery for patients with physical and mental conditions [35]. Conversational agents for health education are necessary because there may be a discrepancy between patients’ actual knowledge needs and what clinical practitioners consider necessary [36-38]. For patients, this digital human serves as a convenient and timely resource tool, providing accurate and personalized information, particularly when clinical staff are unavailable. *Lancet Digital Health* once mentioned the urgent concern regarding whether existing tools and digital health interventions are appropriate for the aging population [39]. In our study, we emphasized this problem and found that the simplicity and convenience of this chatbot bridged this gap based on the results of phase 3 and phase 4. In usability questionnaires and in-depth interviews, almost no patients mentioned that the system was difficult to use.

The design of the nurse avatar and nonverbal behavior received many praises which proved that embodied representation did have significant impacts on patient satisfaction and health outcomes [40]. This was explained in the research by Bickmore et al [40] that human relationships are fundamentally formed in face-to-face conversation and nonverbal behaviors are vital for facilitating social connection and understanding. However, the input and output modalities may require further optimization. Contrary to our expectations, some end users experienced confusion and discomfort when using the voice input to initiate interactions. Patients with limited verbal expression skills or those speaking regional dialects faced difficulties in recognizing their questions accurately. In addition, the chatbot currently provides responses primarily in text format. Incorporating multimedia content, such as images and videos, may further enhance users’ comprehension and reinforce health education.

Health education content should be dynamically adapted to individual patients’ health literacy levels to maximize relevance and effectiveness. Personalization of our conversational agent remains limited. Some conversational agents integrate patients’ appointment schedules and personal health information to deliver more personalized health services. However, the incorporation of private data inevitably amplifies concerns regarding privacy and data protection. Fournier-Tombs and McHardy [41] listed a list of potential ethical risks in conversational chatbots, including discrimination, stereotyping, exclusion, lack of privacy, poor data governance, stigma, error tolerance, and overconfidence. Specifically, our team set safety guardrails to remind patients that clinicians’ judgments should be regarded as the final suggestion in medical decision-making.

In our study, stakeholders were also involved among the 4 phases to provide diverse perspectives on the implementation of our conversational agent in clinical practice. Through quantitative and qualitative research, we found that for medical professionals, especially frontline staff, integrating a domain-specific conversational agent into the gastric cancer clinical workflow amidst growing clinical workloads can streamline patient-clinician communication. By alleviating such burdens, clinicians were allowed to concentrate on more complex tasks, such as observing patient conditions. Moreover, clinical experts also expressed interest in leveraging conversational agents for complex tasks such as data analysis. These findings represent important directions for future development in clinical practice.

Advancing health equity worldwide is increasingly drawing attention in recent years [42]. Although medical chatbots are gaining popularity, there is still a dearth of chatbots specific to gastric cancer health education in resource-constrained regions [43]. Researchers and governments have a responsibility to design and implement fair, unbiased, and easily accessible tools that prioritize the needs of patients [44], and our conversational agent provided a promising solution. Once the health education conversational agent in our study can be put into formal use after effectiveness validation, shared professional knowledge bases will be available for regions’ lack of medical resources, thereby improving the health literacy of local patients, promoting health equity, and eliminating health disparities [45].

### Comparison With Prior Work

Enthusiasm for conversational agents as means for health education was revealed among patients and clinical staff in the first phase of our study, which was also proved in other research [46]. Multiple research studies used theory frameworks to develop such digital interventions. For example, a design science research methodology was used to develop a conversational agent for enhanced self-management after cardiothoracic surgery and verified its acceptability [47]. Similarly, our study adopted an action research approach by realizing the user-centered design and usability principles.

Research gaps remain in gastric cancer–specific chatbots although numerous mature conversational agents have been developed in the health care area. Gomaa et al [9] developed a self-management program in patients with gastrointestinal cancer undergoing chemotherapy and used a mixed methods design to evaluate its effectiveness. However, this platform relied on a structured approach instead of NLP, which limited its understanding of nuanced patient contexts. Zhou et al [15] developed a gastrointestinal disease chatbot using RAG and evaluated its performance with the Retrieval Augmented Generation Assessment framework, which provided an important technical foundation for our study. In contrast to the study by Zhou et al, we enriched our research perspectives by adding qualitative results. Similarly, Kim and Park [48] developed a chatbot for patients with gastric cancer undergoing radical gastrectomy and evaluated both user experience and system performance. The accuracy of our conversational agent is comparable with this similar chatbot. An embodied conversational agent may potentially improve engagement by providing additional motivational and emotional support [49], while also providing a foundation for multimodal interaction [50]. Such design elements remain uncommon in existing health-related conversational agents, especially those targeting gastric cancer care. Besides, an evidence-based corpus, RAG technology, model fine-tuning, and safety guardrail mechanisms were implemented in our study to reduce the risk of generating misleading or unsafe information and improve the reliability of the chatbot’s outputs.

### Limitations and Future Directions

Although the study’s strengths lie in its novel combination of digital health intervention development and action research approach, leveraging both quantitative and qualitative data, some limitations must be acknowledged. This study reported only a single cycle of the action research framework. Additional cycles are necessary to iteratively refine the system and evaluate its effectiveness in real-world settings. Although prior studies suggested that subjective ratings and expert evaluations can provide valuable insights into revealing potential risks in real-world use [51], accuracy rate, RAG knowledge hit rate, and usability score were used to test the performance of the chatbot, which was not enough. The complexity of health care contexts and variability in expert judgment may produce bias and inconsistency [52]. Besides, this study was conducted with a relatively small sample from a specific center and cultural context. Stakeholder perspectives were collected during a limited period, which may not fully capture evolving needs and long-term experience over time. In this study, we focused more on the development and implementation of the conversational agent rather than its clinical effectiveness; the impact of the intervention on patient outcomes and other medical facilities remains unknown without multicenter randomized controlled trials.

Last but not least, algorithmic limitations need further attention. The conversational agent was developed using OpenMEDLab2.0 [31], whose technical description is currently available primarily as a preprint rather than a peer-reviewed publication. Although the model was selected because of its medical domain-specific design, future studies should further evaluate and compare its performance with other peer-reviewed medical foundation models across diverse clinical tasks and settings. Furthermore, the current workflow requires enhancement to support functional extensions, such as follow-up questionnaire delivery. Moreover, the system primarily retrieves answers from a predefined knowledge base without incorporating expert clinical reasoning, highlighting the need for further development of chain-of-thought capabilities.

### Conclusions

This study provided insights into how the action research approach can inform the development and usability assessment of a gastric cancer health education conversational agent. It also illustrated details of how technologies such as RAG, model fine-tuning, and safety guardrail technologies can be integrated into the conversational agent. Promising results regarding accuracy and usability were demonstrated through both qualitative and quantitative research. However, additional assessments and improvements are warranted to confirm the effectiveness, safety, and long-term adoption. Enhancements such as personalization, multimodal data integration, and advanced reasoning capabilities should also be explored in further studies.

## Supplementary material

10.2196/82746 Multimedia Appendix 1 In-depth interview outlines, sample questions, recruitment criteria, evidence resource of the knowledge corpus, detailed results of usability testing, and qualitative results.

10.2196/82746 Multimedia Appendix 2 Electronic materials for conversational agent.

10.2196/82746 Checklist 1 The reporting guideline and checklist “Towards a Checklist for Improving Action Research Quality in Healthcare Contexts.”

## Abbreviations

C/S: Client/Server
CUQ: chatbot usability questionnaire
LLM: large language model
NLP: natural language processing
Q&A: question and answer
RAG: retrieval augmented generation
RLHF: reinforcement learning from human feedback
