# Supplementary material for: Development and Usability Assessment of a Health Education Conversational Agent for Patients With Gastric Cancer: Action Research Study
Source: J Med Internet Res. 2026 Sep 10;28:e82746. doi: 10.2196/82746 (PMC13561191; doi:10.2196/82746)
Supplement: Supplementary file 1 [file jmir-v28-e82746-s001.docx]

**Development and Usability Assessment of a Health Education Conversational Agent for Patients with Gastric Cancer: An Action Research Study**

**Content**

**Table S1. In-depth interview outline with 16 patients and 12 stakeholders prior to the conversational agent development.**

**Table S2. Sample questions randomly selected in the database and numbers of questions of each testing.**

**Table S3. In-depth interview outline with 15 patients and 8 nurses to gather user feedback of the conversational agent.**

**Table S4. Participants and recruitment criteria in each phase.**

**Table S5. Evidence resource of the knowledge corpus.**

**Table S6. Each score of CUQ (rating scale 1-5).**

**Text 1. Detailed analysis of in-depth interview in phase one.**

**Text 2. Detailed analysis of in-depth interview in phase four.**

**Table S1. In-depth interview outline with 16 patients and 12 stakeholders prior to the conversational agent development.**

| Participant group | Key questions |
| --- | --- |
| Patients | 1. Can you describe the health education you have received during your hospital stay? 2. Which parts were difficult to understand or remember? 3. How did you usually seek information when you felt unsure or worried? 4. Did you have any suggestions for the current health education mode? 5. What kind of help would you expect from a digital or conversational tool for health education? |
| Nurses | 1. How do you usually provide health education to patients? 2. What challenges do you encounter when delivering health education? 3. Which topics require repeated explanations? 4. In what ways do you think a conversational agent could support or interfere with your work? |
| Nursing managers | 1. How is health education currently organized in your ward? 2. What gaps or inconsistencies do you observe in current practices? 3. What concerns would you have regarding the introduction of a conversational agent? 4. How should such a tool be integrated into existing workflows? |
| Surgeons | 1. What health education topics do you think are most critical for patients with gastric cancer? 2. What concerns would you have about a conversational agent providing health education? |
| Clinical psychologist and dietitian | 1. How do you currently design and deliver health education materials for patients with gastric cancer in your field? 2. What limitations do you see in traditional health education approaches? |

**Table S2. Sample questions randomly selected in the database and numbers of questions of each testing.**

| Domain | Number of questions | | | Sample questions |
| --- | --- | --- | --- | --- |
|  | 1* | 2** | 3^#^ |  |
| Disease Knowledge | 8 | 10 | 6 | What exactly is this disease, and how does it affect my body? |
| Disease Diagnosis | 5 | 6 | 4 | What are the common symptoms of gastric cancer? |
| Disease Prevention | 5 | 5 | 3 | Which lifestyle habits help reduce the risk of gastric cancer? |
| Disease Treatment | 7 | 8 | 5 | What surgical methods should be selected for different types of recurrent gastric cancer? |
| Nutrition | 6 | 8 | 4 | What should I eat or avoid to support my treatment and recovery? |
| Nursing care | 6 | 7 | 5 | What should be noted regarding home nursing care for patients with gastric cancer? |
| Recovery | 6 | 6 | 4 | What should I do during recovery? |
| Follow-ups | 6 | 6 | 3 | How often do I need follow-up visits, and what will be checked? |
| Hospitalization Procedures | 6 | 6 | 4 | What should I prepare during the hospital admission and discharge process? |
| Total numbers of questions | 55 | 62 | 38 |  |

* Number of questions in first round of alpha testing

** Number of questions in second round of alpha testing

^#^ Number of questions in third round of alpha testing

Note: The number of questions varied slightly across rounds due to the automated sampling process, which selected questions proportionally from different modules of the knowledge base. As each module contains a different number of eligible question–answer pairs, the final number of sampled questions differed across rounds.

**Table S3. In-depth interview outline with 15 patients and 8 nurses to gather user feedback of the conversational agent.**

| Participant group | Key questions |
| --- | --- |
| Patients | 1. Can you describe your overall experience of using the conversational agent for gastric cancer health education? 2. How easy or difficult was it for you to interact with the conversational agent (e.g., asking questions, understanding the responses)? 3. How clear and understandable was the information provided by the agent? Did you feel overwhelmed by the amount of information at any point? 4. What changes or improvements would you suggest to make the conversational agent more helpful or easier to use? |
| Nurses | 1. How do you perceive the usability of the conversational agent when applied in routine nursing practice? 2. How well do you think the conversational agent fits into the existing nursing workflow? 3. Based on your observations, how did patients respond to using the conversational agent for health education? 4. What concerns do you have about using this conversational agent in clinical settings, and what recommendations would you offer for its further development? |

**Table S4. Participants and recruitment criteria in each phase.**

| Phases | Participants | Recruitment criteria |
| --- | --- | --- |
| Phase 1: Diagnose and plan (in-depth interviews) | 16 patients | Adults aged 18 years or older; diagnosed with gastric cancer and having received surgical treatment or perioperative care; had experience with the current health education provided during hospitalization; willing to participate in the study and able to provide informed consent. |
|  | 12 stakeholders (5 nurses, 3 nursing managers, 2 surgeons, 1 clinical psychologist and 1 dietitian) | Nurses: at least 3 years of clinical experience in patient care and education; actively involved in patient education and daily communication with patients with gastric cancer.  Nursing managers: minimum of 10 years of managerial or supervisory experience in nursing or patient education; familiar with hospital policies and patient education workflows, able to advise on program implementation and evaluation.  Surgeons: direct responsibility for gastric cancer treatment and patient management; at least 8 years of clinical experience in gastric surgery.  Clinical Psychologist: Licensed clinical psychologist with at least 3 years of experience in patient counseling; experience in designing or delivering patient-centered educational for cancer patients.  Dietitian: registered clinical nutritionist with at least 3 years of experience in oncology nutrition;  experience in developing patient education materials regarding dietary management during cancer treatment. |
| Phase 2: Act and implement and Phase 3: Evaluate (usability testing) | 28 patients | Diagnosed with gastric cancer and having received surgical treatment or perioperative care; had experience with the conversational agent during hospitalization; willing to participate in the study and able to provide informed consent. |
| Phase 4: Reflect (in-depth interviews) | 15 of patients who finished usability testing | Patients who finished usability testing in last phase. |
|  | 8 clinical nurses | Nurses who worked in the gastrointestinal center and participated in daily health education activity. |

**Table S5. Evidence resource of the knowledge corpus.**

A combination of keywords and Medical Subject Headings (MeSH) terms: “gastric cancer”, “gastric cancer prevention”, “gastric cancer treatment”, “health education”, “patient education”, “nursing care”, “recovery”, “follow-ups”, “nutrition”, “rehabilitation” was used to search the electronic databases. Searches were limited to publications in English or Chinese, with no restriction on publication year. Additional searches included hand-searching reference lists of relevant articles, institutional repositories, and hospital records. Multimedia resources and structured knowledge databases were also queried using topic-relevant keywords aligned with gastric cancer care, nutrition, and patient education.

Multiple sources were included: domestic and international guidelines and consensus statements, scientific literature, monographs, national policies and regulations, hospital structured data, doctor–patient interaction texts, patient feedback, materials from the gastrointestinal surgery studio, multimedia resources, standard medical terminology databases, professional food composition databases, and existing structured knowledge bases. Detailed information was shown in the table below.

| Section | Subsection | Detailed contents |
| --- | --- | --- |
| Official guidelines and professional literature | Domestic guidelines and consensus statements | Chinese Society of Clinical Oncology (CSCO) Clinical Guidelines for Gastric Cancer (2026 Edition), issued by the Chinese Society of Clinical Oncology;  Guidelines for the Clinical Application of Parenteral and Enteral Nutrition in Adult Patients (2023 Edition), issued by the Chinese Society for Parenteral and Enteral Nutrition of the Chinese Medical Association;  Guidelines for Medical Nutrition Therapy in Adult Patients with Gastrointestinal Dysfunction (2025 Edition), issued by the Chinese Society for Parenteral and Enteral Nutrition of the Chinese Medical Association. |
|  | International guidelines and consensus statements | NCCN Clinical Practice Guidelines in Oncology: Gastric Cancer; ESMO Clinical Practice Guidelines: gastric cancer; Korean Practice Guidelines for Gastric Cancer 2024; other guidelines on cancer nutrition, survivorship, rehabilitation, and supportive care issued by ESPEN, ASCO, and other authoritative organizations. |
|  | Scientific literature | Systematic reviews, meta-analyses, high-quality clinical trials, evidence summaries, and qualitative studies on gastric cancer treatment, symptom management, patient education needs, nursing care, nutrition, rehabilitation, quality of life, and survivorship. |
|  | Monographs | Classic textbooks such as Technical Guidelines for Integrated Cancer Diagnosis and Treatment in China (2022), Gastric Cancer (2015), Gastrointestinal Surgery (2025). |
|  | National policies and regulations | Policy documents such as the National Nutrition Plan (2017–2030) issued by the General Office of the State Council, and the Outline of Food and Nutrition Development in China (2025–2030) jointly issued by the National Health Commission, the Ministry of Industry and Information Technology, and the Ministry of Agriculture and Rural Affairs. |
| Clinical data | Hospital structured data | Standardized health education manuals developed by the Clinical Department, disease-related forms and records within clinical pathways, typical therapy protocols and discharge guidance documented in the electronic medical record system. |
|  | Doctor–patient interaction texts | Question-and-answer records of common gastric cancer-related issues from follow-up outpatient clinics and hospitalized patients, records of guidance provided by dietitians, physicians, and nurses, and documentation of typical issues and management strategies from healthcare staff work logs. |
|  | Patient feedback | Records of online consultations with patients, and patient-reported difficulties and symptom feedback collected through follow-up systems. |
| Online resources | Gastrointestinal surgery studio | Frequently asked questions from patients with gastric cancer in the studio. |
|  | Multimedia resources | Educational videos, articles, and other materials on gastric cancer published by professional organizations. |
| Terminology and knowledge system | Standard medical terminology database | Standardized terminology from ICD, WHO Classification of Tumors, Chinese medical terminology standards, and terminology related to gastric cancer diagnosis, staging, treatment, nursing, symptoms, nutrition, rehabilitation, and follow-up. |
|  | Professional food composition database | The Chinese Food Composition Table published by the Chinese Center for Disease Control and Prevention, as well as international food composition databases. |
|  | Structured knowledge base | Structured summaries of gastric cancer–related information from medical knowledge graphs and encyclopedias. |

**Table S6. Each score of CUQ (rating scale 1-5).**

| Positive Scale items | Mean (SD) | Median (IQR) | Negative Scale items | Mean (SD) | Median (IQR) |
| --- | --- | --- | --- | --- | --- |
| The chatbot’s personality was realistic and engaging | 4.5 (0.6) | 5 (4-5) | The chatbot seemed too robotic | 1.4 (0.6) | 1 (1-2) |
| The chatbot was welcoming during initial setup | 4.9 (0.4) | 5 (5) | The chatbot seemed very unfriendly | 1.0 (0.2) | 1 (1) |
| The chatbot explained its scope and purpose well | 4.9 (0.4) | 5 (5) | The chatbot gave no indication as to its purpose | 1.1 (0.3) | 1 (1) |
| The chatbot was easy to navigate | 5.0 (0.2) | 5 (5) | It would be easy to get confused when using the chatbot | 1.0 (0.2) | 1 (1) |
| The chatbot understood me well | 4.1 (0.4) | 4 (4) | The chatbot failed to recognize a lot of my inputs | 2.4 (0.7) | 2 (2) |
| Chatbot responses were useful, appropriate and informative | 4.6 (0.7) | 5 (4-5) | Chatbot responses were irrelevant | 2.1 (0.8) | 2 (2) |
| The chatbot coped well with any errors or mistakes | 4.9 (0.3) | 5 (5) | The chatbot seemed unable to handle any errors | 1.1 (0.3) | 1 (1) |
| The chatbot was very easy to use | 5.0 (0.0) | 5 (5) | The chatbot was very complex | 1.0 (0.0) | 1 (1) |

**Text 1. Detailed analysis of in-depth interview in phase one.**

**1. Fragmented and inconsistent health education content**

**1.1. Lack of continuity across care stages**

In the process of treatment and recovery in gastric cancer care, participants reported that health education was often delivered in a stage-specific and disconnected manner, lacking continuity across different phases of care. Educational content provided during hospitalization primarily focused on surgical procedures and immediate postoperative care, whereas guidance related to long-term symptom management, nutrition, and lifestyle adjustment was insufficiently reinforced after discharge. Patients perceived that health education was fragmented, with limited follow-up or linkage between information delivered at different time points, which hindered their ability to integrate knowledge and apply it consistently throughout the recovery process. As care transitioned from hospital to home, patients frequently experienced gaps in education, leaving emerging questions unanswered and increasing uncertainty in self-management.

*“I felt that the education was different at each stage. What they told me before surgery was not mentioned again after I went home, and I didn’t know who to ask when new problems came up.” (P3)*

*“In the hospital, nurses explained many things, but after discharge, it was like starting over by myself. There was no continuous guidance connecting the hospital care with daily life at home.” (P7)*

**1.2. Overload of information at a single time point**

With the implementation of enhanced recovery after surgery (ERAS) principles, the traditional health education model did reveal certain shortcomings. An overwhelming amount of information was delivered within a short time due to the short time of the health education sessions; patients were limited to absorb and retain key education content. Some patients reported difficulty recalling oral and printed instructions, noting that these methods were inadequate and insufficient in addressing their ongoing and emerging concerns during their care.

*“I listened very carefully when the nurse was giving the health education, but afterward, I could only rely on the printed leaflet they gave me to recall the information……I feel like I didn’t remember a lot of it.” (P1)*

*“When the doctor was explaining, there was just too much information, and they spoke rather quickly. My spouse and I are getting older now, and it's hard for us to memorize everything.” (P5)*

**2.Limited accessibility and personalization of education delivery**

**2.1. Time constraints in face-to-face education**

Participants consistently reported that face-to-face health education was constrained by limited time and heavy clinical workloads, which restricted the depth and personalization of educational interactions. Nurses and physicians often delivered key information under time pressure, focusing on essential instructions rather than patients’ individual concerns or comprehension levels. As a result, patients felt hesitant to ask follow-up questions and perceived that some important details were overlooked. This time-limited model reduced opportunities for clarification and reinforcement, particularly for patients who required repeated explanations due to emotional stress or limited health literacy.

*“The nurses were very busy, and I could tell they didn’t have much time. They explained the main points quickly, but I didn’t want to keep asking questions because they seemed rushed.” (P2)*

*“Sometimes I still had doubts, but the doctor had to leave for the next patient, so the education felt a bit incomplete.” (P6)*

**2.2. One-size-fits-all educational materials**

Many participants noted that existing health education materials were standardized and insufficiently tailored to individual patient needs, disease stages, or personal circumstances. Printed leaflets and routine verbal instructions were often generic, providing broad information that did not adequately address patients’ specific symptoms, treatment plans, or cultural and educational backgrounds. This uniform approach limited the relevance and usefulness of the educational content, making it difficult for patients to identify which information was most applicable to their own situation.

*“The booklet they gave me was the same for everyone. Some parts didn’t apply to me at all, while the things I really cared about were not explained in detail.” (P4)*

*“I felt the education was very general. Patients like us have different problems, but the materials were all the same.” (P8)*

**2.3. Difficulty revisiting information after discharge**

After discharge, patients frequently experienced difficulty accessing and revisiting health education information provided during hospitalization. Verbal instructions were easily forgotten, and printed materials were often misplaced or insufficient to address new or evolving concerns. Participants expressed a strong need for a convenient and reliable way to review information when questions arose at home, particularly regarding symptom management, diet, and activity. The lack of ongoing access to professional guidance increased patients’ uncertainty and reliance on potentially unreliable external information sources.

*“Once I was discharged, I couldn’t remember many of the things they told me in the hospital. When questions came up, I didn’t know where to find accurate answers.” (P1)*

*“At home, problems appeared that weren’t discussed before, and there was no easy way to go back and check the information.” (P9)*

**3.Unmet needs for timely and interactive support**

**3.1. Delayed responses to emerging concerns**

Participants described experiencing new or intensified concerns at various points during the treatment trajectory, particularly in the perioperative and post-discharge phases. While initial health education addressed some anticipated questions, it was often insufficient to meet patients’ evolving informational and emotional needs. Due to limited access to healthcare professionals outside scheduled encounters, patients reported delays in obtaining timely responses to emerging concerns. This delay not only heightened anxiety but also discouraged proactive help-seeking, as patients worried about imposing additional burdens on already busy clinicians.

*“I was very anxious before the surgery……Although the preoperative education helped alleviate some of my anxiety and answered some of my questions, I felt it wasn't enough. However, I was also worried about bothering the doctors and nurses too much.” (P3)*

**3.2. Reliance on informal or online information sources**

In the absence of timely professional guidance, many patients turned to informal channels or online sources to seek health information. Participants reported searching the internet, social media platforms, or consulting peers with similar experiences to address unanswered questions. However, they also expressed concerns about the credibility and consistency of such information, noting that conflicting or overly alarming content often increased confusion and anxiety. This reliance on unverified sources highlighted a gap between patients’ information needs and the support provided by formal health education systems.

*“When I couldn’t get answers from the hospital, I searched online. But the information was very mixed, and sometimes it made me more nervous.” (P5)*

*“I asked other patients or read posts online, but I wasn’t sure which advice was reliable.” (P10)*

**3.3. Desire for repeated and on-demand explanations**

Participants emphasized a strong need for repeated and easily accessible explanations of health education content. Due to emotional stress, physical discomfort, and information overload, a single explanation was often insufficient for effective understanding and retention. Patients expressed a preference for on-demand access to educational support that would allow them to revisit information at their own pace and seek clarification whenever needed. Repetition and availability were viewed as essential for building confidence in self-care and decision-making throughout the disease trajectory.

*“Sometimes I needed the same information explained again, especially when I was feeling unwell. One explanation just wasn’t enough.” (P7)*

*“I hoped there was a way to ask questions anytime and get clear answers, instead of waiting for the next hospital visit.” (P2)*

**4.Expectations and concerns for new, technology-supported education manners**

**4.1. Continuous and on-demand access to education**

Patients showed positive attitude to intelligent health education conversational agent. While the traditional health education approach had helped improve patients’ health literacy and knowledge, many still expressed a desire for more personalized, accessible, and easier-to-understand educational resources. This need is particularly salient among perioperative patients, as comprehensive health education has the potential to reduce anxiety and depressive symptoms during their surgical journey.

*“The current health education has indeed provided me with a lot of new information, but I still hope for more diverse forms of health education.” (P15)*

*“……If there were an intelligent robot, I think it would be very helpful to me. It could repeat the health education content for me, so I wouldn’t have to keep bothering the doctors and nurses with repeated questions, which would also increase their workload.” (P10)*

**4.2. Interactive and conversational learning experience**

Participants expressed a clear expectation for health education to move beyond one-way information delivery toward a more interactive and conversational learning experience. Traditional education methods were perceived as passive, limiting opportunities for engagement, clarification, and personalization. In contrast, patients envisioned an intelligent and responsive approach that could actively interact with them, adapt explanations based on their questions, and provide timely feedback according to their individual needs and disease stages. Such an interactive model was perceived as more aligned with the modern information environment and more capable of supporting continuous learning, emotional reassurance, and informed self-management.

*“We’re living in the information age now, and I hope there can be more intelligent methods and approaches for delivering health education to me.” (P6)*

**4.3. Trustworthy and professionally validated information**

Stakeholders also expressed a positive attitude toward integrating digital technologies with patient education. They believed such integration could not only enhance patient satisfaction but also reduce the workload of clinical staff and health educators.

*“If there were such a machine, I would be truly delighted. It could be a great assistant in my work.” (S5)*

*“I really think we need a machine or device now to help take on part of the health education work, because sometimes patients have a lot of questions, and they're quite different. I'm either repeating myself over and over, or I simply don't know the answers.” (S1)*

However, they also emphasized the need to ensure that any digital platform delivers information should be accurate, safe, and ethically sound.

*“……Although having such a device could reduce our workload, after all, it’s designed for patients, so safety must be ensured—otherwise, it could lead to unnecessary risks.” (S4)*

*“AI has indeed developed rapidly and is widely applied now, but we must also use it with caution. It should only be introduced to patients after confirming that it is reliable, practical, and provides accurate responses.” (S8)*

**Text 2. Detailed analysis of in-depth interview in phase four.**

**Domain 1: Perceived usefulness-Personalized, timely, and accessible support; Information and content presentation limitations; Workload reduction and information omission prevention**

The application of conversational agent among patients reflected the characteristics of personalization, accessibility, and patient-centeredness. Most patients reported that this chatbot provided them with timely and relevant health information during their hospitalization, greatly improving their understanding of their condition, especially during different periods before and after surgery. They also appreciated the function that they could repeatedly ask questions according to their needs and receive timely guidance. Some patients particularly emphasized the value of this conversational agent when there was “no one to ask,” such as at night or when clinical staff were not available, which helped alleviate anxiety and unease.

*“It was like having a knowledgeable companion by my side who could answer my questions at any time.” (T3)*

*“I was lying awake at night, a little worried about what and how to eat after I was discharged, so I asked the robot, and it told me what I could and couldn't eat, which put my mind at ease.” (T5)*

However, some patients suggested that the platform designed for the conversational agent was too limited, and the information provided was too complex and not concise enough.

*“Sometimes I asked a very simple question, but the answer is too long and the language is quite academic, which in turn made me feel burdened.” (T7)*

The conversational agent had also greatly reduced the workload of clinical staff and the time spent on repetitive health education. Some employees also said that the chatbot's rich knowledge base ensured that health education content is comprehensive and accurate, preventing the omission of key information due to manual education.

*“Patients often asked the same questions repeatedly. In our busy daily treatment work, if we have a conversational agent, we can save this time and spent more time to observe the patient's condition or other more important matters.” (N1)*

*“We may not always be able to provide patients with comprehensive educational content, but the conversational agent helps us to supplement this and make up for any omissions in our work.” (N2)*

**Domain 2: Perceived ease of use-Intuitive interface and multi-modal functionality; Recognition barriers and dialect challenges; Operational simplicity and future intelligent integration demand**

The characteristics of the chatbot's usability shown in the usability questionnaire were also reflected in the patient interviews. Most patients reported that the chatbot was easy to operate, had a clear interface, and had a voice narration function, allowing many elderly patients to quickly learn how to use it with simple instructions.

*“I thought it would be difficult to use, but after the nurse showed me how, I learned it immediately. It wasn't difficult at all.” (T10)*

*“The machine read out the answers and allowed you to adjust the sound. I think it's much more useful than paper materials.” (T14)*

Although voice functions have alleviated some of the difficulties patients have in reading text, for some patients who speak dialects or have language impairments, conversational agents may report errors or fail to recognize, thereby rendering health education conversational agents unusable.

*“Do I have an accent? So when I use it, it always misunderstood what I'm saying, which seems to affect its usability a little.” (T8)*

Clinical workers also recognized the ease of use of this conversational agent, but in order to better improve the utilization of the system, some employees suggested that we should add more functions.

*“This conversational agent is indeed very convenient to use, and we don't really need to teach patients how to use it.” (N5)*

*“I hope this machine can integrate data analysis or other more intelligent functions, not just for patient health education, but also for other purposes.” (N3)*

**Domain 3: Intention to use-Humanized design and emotional connection; Desire for post-discharge use via mobile applications; Continuous updates and management-level optimizations**

Patients highlighted the use of a nurse avatar, which they felt enhanced the sense of empathy and professionalism. This humanized design helped to build emotional connection and increased their willingness to engage with the tool.

*“It feels like I’m really talking to a nurse, not just a machine……She looks kind and makes me feel more at ease when asking questions.” (T9)*

Most patients expressed a desire to continue using the service during their hospital stay and at home, and hoped that a mobile app could be developed in the future to make it easier to use this health education chatbot.

*“I feel that this machine is really easy to use. It would be great if you could develop a mobile app so that I can ask questions when I get home if I don't understand something.” (T6)*

Nursing staff expressed their willingness to continue using the tool in their daily work and recommend it to patients, especially those undergoing gastric cancer surgery for the first time. They also believed that the knowledge base should be continuously updated to ensure scientific accuracy and rigor. Some managers suggested adding analytical functions in the future, such as statistics on frequently asked questions from patients, to help optimize the focus of education and outreach.

*“This conversational agent is very useful, but we still need to improve it to make it more mature and reliable.” (N1)*

*“I hope it can add or refine more functions to cover more of the repetitive low-level tasks we do on a daily basis.” (N3)*
