# Supplementary material for: Development and Usability Assessment of a Health Education Conversational Agent for Patients With Gastric Cancer: Action Research Study
Source: J Med Internet Res. 2026 Sep 10;28:e82746. doi: 10.2196/82746 (PMC13561191; doi:10.2196/82746)
Supplement: Supplementary file 2 [file jmir-v28-e82746-s003.docx]

Casey, et al. Towards a Checklist for Improving Action Research Quality in Healthcare Contexts. *Systemic Practice and Action Research* (2023) 36:923–934 <https://doi.org/10.1007/s11213-023-09635-1>

**Checklist QuARC**

| **Factors** | **Questions** | **Section** |
| --- | --- | --- |
| Context | 1. Is the action driven by a practical concern? | Background |
|  | 2. Is there local, national, and international imperative influencing the practical concern? | Background |
|  | 3. Does previous research inform the practical concern? | Background |
|  | 4. Does the AR project draw on previous research? | Background & Discussion |
| Quality of relationships | 1. Are those who have articulated the practical concern in the first instance and who also have a stake its resolution included in all the steps of the action research cycle of constructing, planning action, taking action and evaluating? | Method |
|  | 2. Can the participants be classified as co-researchers? | Method |
|  | 3. Is the level of participation of the patients/clients and coresearchers evident at each step of the action research cycle? | Method |
|  | 4. Is the AR project evaluated in terms of the quality of the relationships? | / |
| Quality of the action research process itself | 1. Does the final account demonstrate a rigorous and collaborative engagement in the action research project’s design? | Result |
|  | 2. Does the account demonstrate subsequent enactment of cycles of constructing, planning action, taking action and evaluating? | Discussion |
|  | 3. Is enactment of the cycle towards the practical and theoretical objectives transparent and reliable? | Method |
|  | 4. Is the engagement of the coresearchers evident in shared data analysis and shared reflection? | Method & Result |
| Outcomes | 1. Are both forms of outcomes presented (theory (research) and action (practical)? | Result |
|  | 2. Are the outcomes sustainable? | Result &  Discussion |
|  | 3. Are the outcomes useful for other action research projects? | Discussion |
|  | 4. Does the theoretical outcome contribute to future theory development? | / |
|  | 5. Is there a reflection on the process of engagement on the cycles by the co-researchers? | Result |
